# Citrate of Iron

**Published:** 1848-10

**Authors:** 


*Citrate of Iron.*—Our friend Edward Parish has shown us a *syrup of citrate of iron*, which appears to be a good preparation. He first prepares a moist protocarbonate of iron, by mixing together solutions of sulphate of iron and carbonate of soda, precisely as directed for Vallet's ferruginous mass, and washing with sweetened water. This is then dissolved by means of a slight excess of citric acid in water, and evaporated to dryness. A greenish, deliquescent, freely soluble, uncrystallizable salt results, the taste of which is ferruginous, but not very unpleasant. To make the syrup, one ounce (troy) of this salt is dissolved in five fluid ounces of simple syrup, which is easily effected, and forms a dark greenish-brown liquid. The dose is from thirty drops to a teasponful. The syrup of citrate of iron of Beral is a saccharine solution of the citrates of ammonia and sesquioxide of iron.—*Journ. of Pharmacy.*

